# Antioxidant Activity of New Aramide Nanoparticles Containing Redox-Active *N*-phthaloyl Valine Moieties in the Hepatic Cytochrome P_450_ System in Male Rats

**DOI:** 10.3390/molecules17078255

**Published:** 2012-07-10

**Authors:** Hammed H. A. M. Hassan, Sabah G. El-Banna, Amel F. Elhusseiny, El-Sayed M. E. Mansour

**Affiliations:** 1 Department of Chemistry, Faculty of Science, Alexandria University, P. O. Box 2-Moharram Beck, Alexandria 21598, Egypt; 2 Department of Analytical and Pharmaceutical Chemistry, Faculty of Pharmacy, Pharos University, Canal El Mahmoudia Street, Alexandria 21311, Egypt; 3 Department of Environmental Studies, Institute of Graduate Studies and Research, Alexandria University, Alexandria 21526, Egypt

**Keywords:** aramide nanoparticles, thermal analysis, valine, cytochrome P_450_ enzymes, TBARS

## Abstract

We report the synthesis of aramide nanoparticles containing a chiral *N*-phthaloyl valine moiety and their antioxidant activities on hepatic contents of cytochrome P_450_, amidopyrene *N*-demethylase, aniline-4-hyroxylase and induced the hepatic content of cytochrome b5 and nicotinamide adenine dinucleotide phosphate (NADPH) cytochrome C-reductase. Polymers were obtained as well-separated spherical nanoparticles while highly aggregated particles via H-bonding organization of the aramide-containing pyridine led to a thin layer formation. The effects of the nanoparticles and CCl_4_ on enzyme activities and thiobarbituric acid reactive substances (TBARS) levels of male rat liver were studied. Pretreatments of rats with the polyamides prior to the administration of CCl_4_ decreased the hepatic content of the tested enzymes. Doses reduced the toxic effects exerted by (•CCl_3_) upon the liver through inhibition of the cytochrome P_450_ system. Inhibition of such metabolizing enzymes could reduce the carcinogenic effects of chemical carcinogens.

## 1. Introduction

The liver plays a major role in regulating various physico-chemical functions of the body, including synthesis, secretion and metabolism of xenobiotics. Liver diseases are amongst the most serious health problems in the World today and their prevention and prevention options still remain limited despite the tremendous advances in modern medicine. The liver, unique in its capacity for regeneration following injury, may give rise to malignancy states of advanced fibrosis or cirrhosis [1,2]. Many etiological factors may induce liver damage, including infectious agents and hepatotoxic chemicals. Carbon tetrachloride (CCl_4_) is one of the most commonly used hepatotoxins for inducing liver injury in experimental animal studies [3]. Various studies have demonstrated that CCl_4_ intoxication causes free radical generation in the liver [4,5,6]. CCl_4_ intoxication in animals is an experimental model that mimics oxidative stress in many physiological situations. The initial step in the tissue injury induced by carbon tetrachloride is its cytochrome P450-mediated transfer of a single electron to the C–Cl bond, giving a radical anion as a transient intermediate that eliminates chlorine to form a carbon centered radical, the trichloromethyl radical (•CCl_3_) and chloride radical [7,8]. The trichloromethyl radical can dismutate to chloroform, bind to macromolecules or attack polyenoic fatty acids in cellular membranes. The double allylic hydrogen atoms in these acids are particularly susceptible to abstraction by free radicals, giving chloroform and secondary lipid radicals which react rapidly with molecular oxygen to form lipid peroxy radicals. The trichloromethyl radical can also react with oxygen to form the peroxy trichloromethyl free radical (•CCl_3_O_2_), which is more reactive than the (•CCl_3_) and produces similar kinds of damage.

Hepatic drug-metabolizing enzyme [9] is called mixed-function oxidase or monooxygenase and contains many enzymes, including phase I enzymes such as cytochrome P-450, cytochrome b_5_ and NADPH-cytochrome C reductase which metabolize most carcinogens and xenobiotics into less and/or more active intermediates. In addition to the above enzymes agents involved in phase II drug metabolism, e.g., glutathione and glutathione S-transeferase, as well as free radicals “detected as thiobarbituric acid-reactive substances, TBARS”. It has been postulated that the rate-limiting step in the oxidation of some xenobiotics may be due to the rate of reduction of cytochrome P450-substrate complex which is dependent on the activation of NADPH- cytochrome C-reductase and of cytochrome P_450_. NADPH-cytochrome C-reductase activity is a component of the microsomal mixed-function oxidase system which catalyses hydroxylation reaction and this process are of a prime importance in the metabolism of lipids, drugs and other foreign compounds. A wide variety of compounds have the ability to increase or inhibit the drug metabolism by their effect on the activity of NADPH-cytochrome C-reductase. The rate-limiting step in the activation and detoxification of toxic compounds is dependent on the rate of reduction of cytochrome P-450-substerate complex, which in turn is dependent on the activation and turnover rates of NADPH-cytochrome C-reductase, cytochrome b_5_ and on the total cytochrome P-450 content.

Antioxidants protect the human body against free radical attacks that may cause pathological conditions. Various compounds with differential antioxidant properties are found in natural resources which are considered to have high potential in the context of therapeutic approaches to encounter and prevent free radical damage [10]. Many plants contain substantial amounts of antioxidants such as vitamins C and E, carotenoids, flavonoids and tannins that can be utilized to scavenge excess free radicals from the human body [11]. The free radical scavenging potential of natural antioxidants varies among diseases and types of antioxidant [12]. Nowadays, there is an increased interest in finding other synthetic products for effective uptake of antioxidant compounds as an alternative source of scavenging reactive oxygen species (ROS) which includes the superoxide anion O_2_, H_2_O_2_ and the hydroxyl radical.

**Scheme 1:**
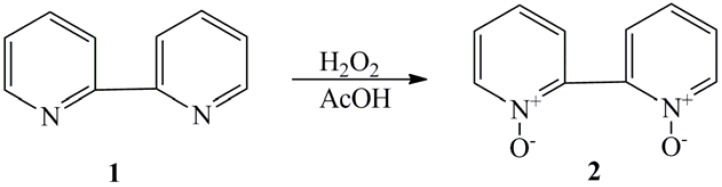
Oxidation of pyridine to pyridine-*N*-oxide.

Aromatic polyamides have excellent thermal stability, good chemical resistance, mechanical strength, and low dielectric constants [13,14]. They are used widely in the microelectronic, aviation, liquid crystal display, and separation industries. However, aromatic polyamides are difficult to process because they are insoluble in most organic solvents and do not flow below their decomposition temperatures. The introduction of flexible chains into polyamide backbone [15], the use of meta-oriented monomers [16,17,18], synthesis of polyamides with non coplanar units in the polymer chains [19], or introduction of bulky side groups into the polymer chains, [20,21,22,23] resulted in a number of modified polyamides. These modifications work by breaking chain symmetry and regularity and by destroying hydrogen bonding, generally leading to better solubility and process-ability. The imine structure on pyridines **1** can be oxidized by 30% aqueous H_2_O_2_ in acetic acid to form pyridine N-oxides **2**, Scheme 1 [24].

Based on this mechanism, we describe for the first time the synthesis and characterization of nanoscale aramides containing *N*-phthaloyl valine moieties in the side chain by a precipitation polymerization method [25,26,27]. Phthalimides are among the most useful organic compounds in biology and synthetic chemistry [28]. The effects of the prepared aramides nanoparticles as antioxidants on the hepatic content of cytochrome P_450_ and other dependent drug-metabolizing enzyme activities were assessed. 

## 2. Results and Discussion

The main objective of this work was to synthesize and characterize aramide nanoparticles containing the chiral *N*-phthaloyl valine moieties and to assess them as antioxidants on the hepatic content of cytochrome P_450_ and other dependent drug-metabolizing enzyme activities. Aromatic polyamides are thermally stable polymers with an attractive combination of excellent chemical, physical and mechanical properties [10]. One of the approaches to increase solubility and processability without sacrificing their thermal stability is the introduction of asymmetric units into the polymer backbone [29,30,31]. Introduction or the attachment of such pendent groups in the polymer main chain imparts a significant increase in T_g_ by restricting the segmental mobility and providing a good solubility as a result of decreased packing and crystallinity. Recent advances in asymmetric reactions and catalysis as well as in chiral separations have afforded a rapid increase in the number of commercially available optically active compounds and reagents [32,33] and the simplest method of synthesizing optically active polymers involve the polymerization of optically active monomers. 

### 2.1. Synthesis of Aramides NanoparticlesContaining N-Phthaloyl Valine Moieties

**Scheme 2:**
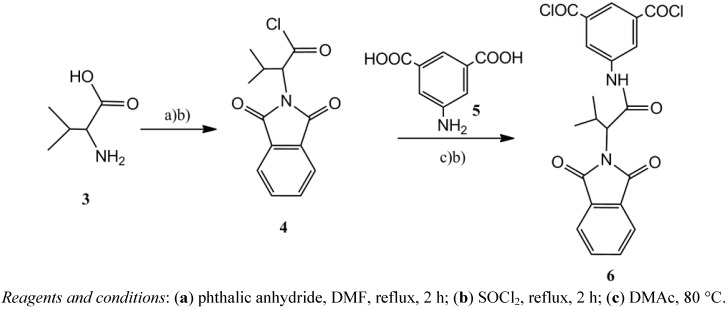
Preparation of 5-(2-phthalimidyl-3-methylbutanoylamino)-isophthaloyl chloride **6** from *S*-valine **3**.

The known 2-phthalimidyl-3-methyl butyric acid chloride (**4**, Scheme 2) [34] was prepared by heating equimolar ratios of *S*-valine (**3**) and phthalic anhydride in DMF, followed by refluxing of the resulting crude material with thionyl chloride. Reaction of **4** with 5-aminoisophthalic acid (**5**) in DMAc smoothly furnished the known 5-(2-phthalimidyl-3-methyl butanoylamino)isophthalic acid. Heating the latter with thionyl chloride followed by co-evaporation of the product with *n*-hexane gave the required diacid chloride **6** in good yield. 

Our attention focused next on the synthesis of new types of chiral aramide nanoparticles. Polymeric aromatic nanoparticles can be prepared by either emulsion or interfacial polymerizations. Additionally, a popular method used for polymeric nanoparticles preparation is solvent displacement, also referred to as nanoprecipitation [35]. The basic principle of this technique is based on the interfacial deposition of a polymer after displacement of a semipolar solvent, miscible with water, from a lipophilic solution. Rapid diffusion of the solvent into the non-solvent phase results in a decrease of interfacial tension between the two phases, which increases the surface area and leads to the formation of small droplets of organic solvent. The key variables determining the success of the method and affecting the physicochemical properties of nanoparticles are those associated with the conditions of adding the organic phase to the aqueous phase, such as organic phase injection rate, aqueous phase agitation rate, the method of organic phase addition and the organic phase to aqueous phase ratio. Likewise, nanoparticle characteristics are influenced by the nature and concentration of their components [36,37]. The process of particle formation in the nanoprecipitation method comprises three stages: nucleation, growth and aggregation. The rate of each step determines the particle size and the driving force of these phenomena is the ratio of polymer concentration over the solubility of the polymer in the solvent mixture. The separation between the nucleation and the growth stages is the key factor for uniform particle formation [38].

**Scheme 3:**
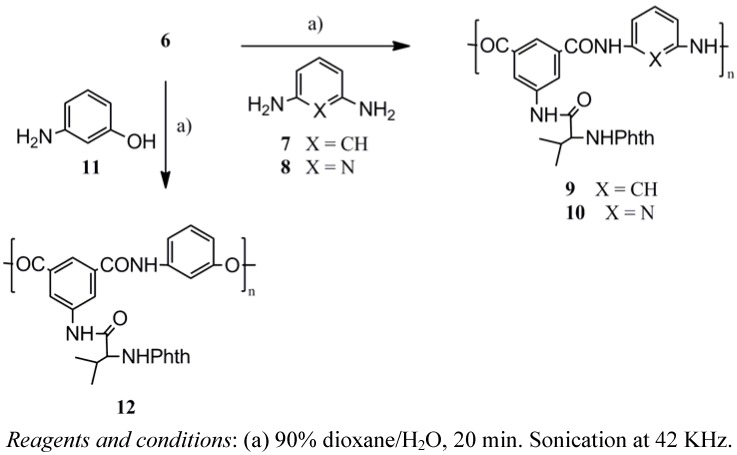
Chemical preparation of polyamide nanoparticles **9**, **10** and the polyesteramide **12**.

**Scheme 4:**
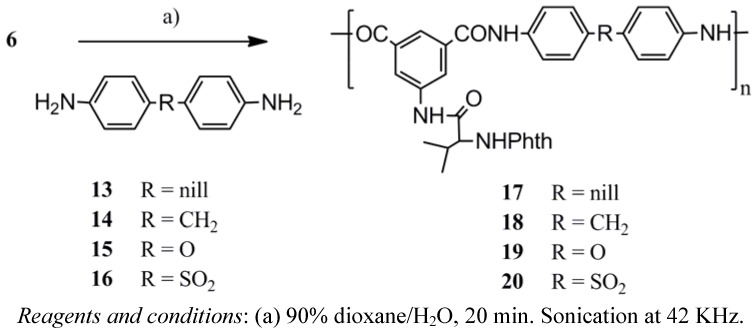
Chemical preparation of polyamide nanoparticles **17**-**20**.

The polyamides nanoparticles **9**, **10** and the polyesteramide **12** (Scheme 3) were prepared by ultrasonication of 0.5 mmol of *m*-phenylenediamine (**7**), 2,6-diaminopyridine (**8**) and *m*-aminophenol (**11**), respectively, with 0.5 mmol of the acid chloride **6** in a total of 115 mL dioxane solution containing distilled water (15 mL) (*i.e.*, 50/15 mL dioxane-water *v/v* diamine solution and 50 mL dioxane acid chloride solution) followed by centrifugal separation at 15,000 rpm for 30 min. Similarly, polyamide nanoparticles **17**–**20** were obtained from the reaction of the acid chloride **6** with benzidine (**13**), 4,4'-diaminodiphenylmethane (**14**), 4,4'-diaminodiphenylether (**15**) and 4,4'-diaminodiphenyl-sulfone (**16**), respectively, under ultrasonication conditions (Scheme 4). Mixing 1,4-dioxane with H_2_O was essential for many reasons, such as controlling the particle morphology, playing an important role in determining the polarity of the reaction solution and as a reaction accelerator [24,25,26,27]. The polymer structures **9**, **10**, **12** and **17**–**20** were confirmed by elemental analysis, IR and UV spectroscopy (Table 1).

**Table 1:** Physical properties of polymers **9**, **10**, **12** and **17**–**20**.

No.	Yield (%)	Unit Formula	M.Wt	% C (Exp.)	% H (Exp.)	% N (Exp.)	η_inh_^a^	IR (KBr) (υ cm^−1^)	λ_max_ (nm)
**9**	88	C_27_H_24_N_4_O_6_	500	64.79 (64.31)	4.83 (4.42)	11.19 (11.38)	0.70	3464, 2968, 1768, 1712, 1608, 1549, 1488, 1450, 1386, 1334, 1248, 1115, 1071, 1017, 886, 686.	265 300
**10**	81	C_26_H_23_N_5_O_6_	501	62.27 (62.63)	4.62 (4.88)	13.97 (13.54)	0.62	3448, 1768, 1712, 1608, 1554, 1488, 1450, 1387, 1333, 1245, 1115, 1071, 1016, 999, 959, 886, 868.	265 305
**12**	86	C_27_H_23_N_3_O_7_	501	64.67 (64.30)	4.62 (4.22)	8.38 (8.09)	0.67	3423, 2966, 1769, 1713, 1656, 1605, 1548, 1494, 1451, 1385, 1354, 1335, 1255, 1174, 1157, 1117, 1077, 1016, 999, 975, 888.	265 296
**17**	90	C_33_H_28_N_4_O_6_	576	68.74 (69.01)	4.89 (4.55)	9.72 (9.37)	0.61	3457, 2967, 1769, 1713, 1652, 1597, 1523, 1502, 1467, 1447, 1415, 1385, 1331, 1116, 1070, 887.	269 306
**18**	84	C_33_H_28_N_4_O_7_	592	66.88 (66.49)	4.76 (4.41)	9.45 (9.78)	0.60	3452, 2967, 1769, 1714, 1655, 1602, 1539, 1499, 1467, 1447, 1408, 1385, 1335, 1232, 1169, 1117, 1071, 1014, 876, 832.	268 292
**19**	88	C_34_H_30_N_4_O_6_	590	69.14 (70.45)	5.12 (5.53)	9.49 (9.81)	0.63	3460, 1713, 1649, 1533, 1411, 1385, 1253, 1071, 720, 531.	268 295
**20**	86	C_33_H_28_N_4_O_8_S	640	61.87 (62.34)	4.41 (4.19)	8.75 (8.42)	0.58	3468, 1770, 1714, 1674, 1593, 1529, 1447, 1394, 1320, 1252, 1148, 1106, 1071, 888, 836.	270 305

^a^ The inherent viscosity of the polymers was measured at a concentration of 0.5 g/dL in DMSO at 30 °C.

**Figure 1:**
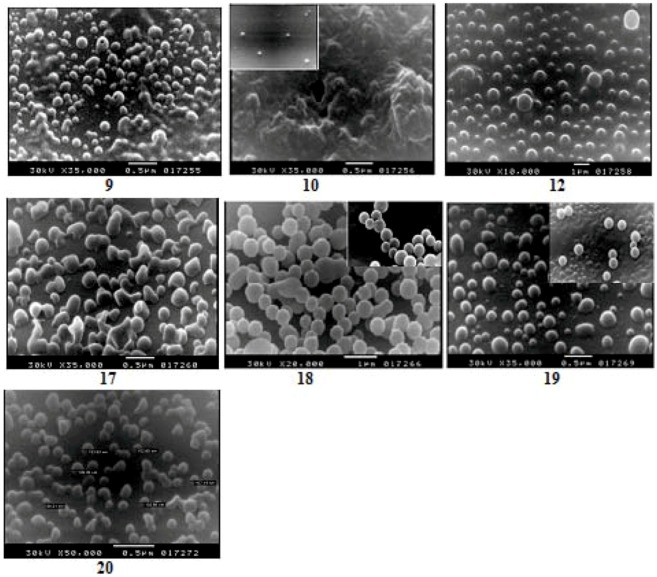
SEM images of the aramides nanoparticles **9**, **10**, **12** and **17**–**20**.

As judged by SEM photographs, (Figure 1), polyamides nanoparticles **9** and **10** were obtained with average diameters of 106 nm, while polyesteramide nanoparticles **12** produced under the same reaction conditions were obtained with larger diameters (190–300 nm). The average diameter of particles was estimated from SEM images and selected at random. Noteworthy, the polymeric particles **9** and **12** were obtained as well-separated spherical nanoparticles while aggregated particles of the aramide-containing pyridine **10** formed a thin layer. The formation of such a thin layer may be attributed to the molecular self assembly via H-bond directed organization of molecular precursors [39]. Amides embody self-complementary recognition groups defined by homomeric H-bond donor-acceptor pairs [40].

**Figure 2:**
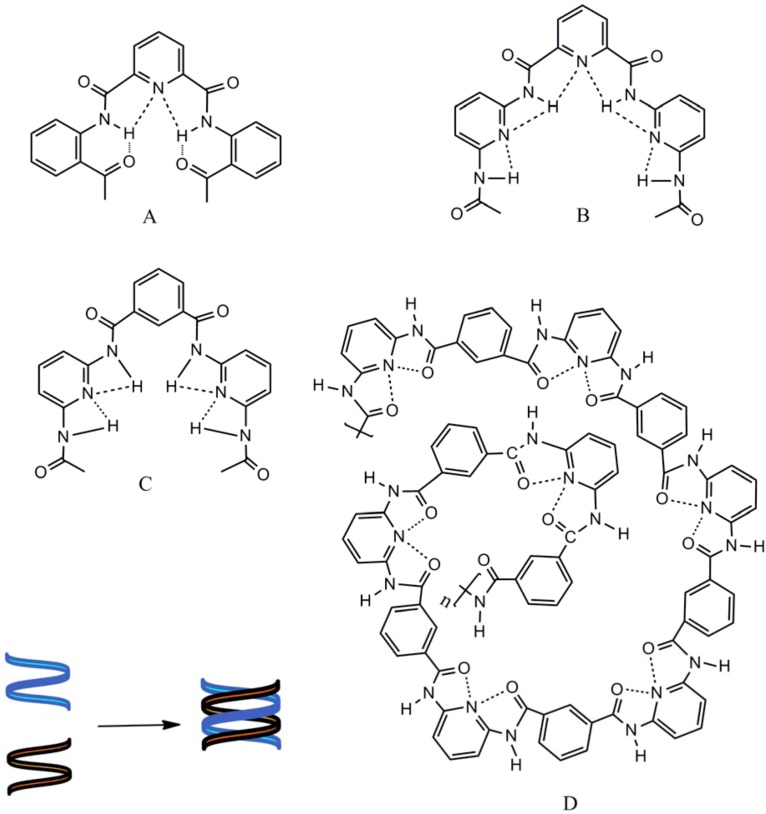
Intramolecular hydrogen bonds in helical conformations of pyridine-containing polymers and schematic representation of double helix made by interactions of two helical monomers.

Aramides containing pyridine assume extended helical secondary structures able to dimerize in solution, undergoing dynamic exchange between single and double helical states. Internal H-bonding, in the form of motifs A and B (Figure 2), induces curvature into the oligomer backbone [41,42]. Similarly, pyridine-containing polyamides **10** assemble through the action of H-bonds according to motifs C or D (Figure 2). In addition, synthetic polymers may also assemble by virtue of homo- or heteromeric association with other molecular strands to yield polymeric duplex or triplex ensembles. In principle, H-bonding could occur in a frame-shifted sense, resulting in the formation of polymeric aggregates. Although the H-bond donor acceptor sites of the composite strands are not in direct juxtaposition, they nevertheless act in concert as a composite H-bonding recognition group. Single strands that reside in well-defined conformations may adopt alternative forms upon complexation with a complementary strand. Figure 2 represents the intramolecular hydrogen bonds in helical conformations of pyridine-containing polymers and schematic representation of double helix made by interaction of two helical monomers [43].

Nanoparticles **17** and **20** were obtained with average diameters of 106 nm and 120 nm, respectively; while nanoparticles **18** and **19** produced under these conditions have larger diameters (230–300 nm). Noteworthy, addition of particular amount of water (15% v/v) to this reaction system is essential, not only for the formation of spherical particles, but also to diminish the aggregation of these particles. The tendency of spherical particles formation of such aramides may be correlated to the dispersion stability of particles in the reaction solution or the precipitation mechanism of the particles. The formation mechanism of polymers is related to the effect of water on the micelle structure. Micelles grow in the presence of water to spherical micelles [25,26,27]. 

### 2.2. Physical Properties of the Prepared Polymers

#### 2.2.1. Solubility

The prepared pyridine-containing polymers **11**–**16** showed different solubility behaviors in different organic solvents. Moderate to complete solubilities (5 wt% solid content) were observed in a variety of hot aprotic solvents such as NMP, DMSO, DMAc and in hot conc.H_2_SO_4_, while they were insoluble in boiling alcoholic solvents such as methanol, ethanol, propanol and ethylene glycol, in halogenated solvents such as CHCl_3_, CCl_4_, CH_2_Cl_2_, ClCH_2_CH_2_Cl or in ethers such as Et_2_O, THF, 1,4-dioxane or 1,2-dimethoxyethane (DME). 

#### 2.2.2. Inherent Viscosity

The inherent viscosity of the polymers, as a suitable criterion for evaluation of molecular weight, was measured at a concentration of 0.5 g/dL in DMSO at 30 °C. It was in the range of *η*_inh_ 0.57–0.70 dL/g that showed moderate molecular weights (Table 1). 

#### 2.2.3. FTIR Spectroscopy

The FT-IR spectra of the polymers exhibited characteristic absorption bands at around 3,300 and 1,769, 1,712 and 1,650 cm^−1^ corresponding to the N–H and C=O stretching of amide groups, respectively, while the bands at around *υ* 3,050, 2,960 and 1,600 cm^−1^ are due to H–C_str_ and C–C_str_, respectively. Table 1 compiles selected IR bands of the polymers **9**, **10**, **12** and **17**–**20**. 

#### 2.2.4. Optical Properties

The optical properties of the prepared polymeric nanoparticles were investigated by UV-Vis spectroscopy in DMSO with a polymer concentration of ~2 mg/10 mL (Table 1). All polyamides exhibited maximum absorptions (*λ*_max_) around 300 nm and 265 nm due to the *n*–π* and *π*–π* transitions, respectively. 

#### 2.2.5. Thermal Properties

The thermal properties of the prepared polymers were evaluated by differential thermo gravimetric (DTG), differential thermal analysis (DTA) and differential scanning calorimetry (DSC) techniques. Thermal data of the prepared polymers are complied in Table 2. Thermal results revealed that the prepared polymers have high thermal stability. Structure-thermal property correlation based on changing the diamine monomer, as a single structural modification, demonstrated an interesting connection between a single change and the thermal properties. Polyamides **9**, **10** and **17**–**20** exhibited major degradation processes at 425 °C leaving 41.40%, 41.81%, 32.00%, 53.18%, 59.02% and 47.71%, respectively, as char yields. Polyesteramide **12** exhibited less thermal behavior and showed a major degradation step at 320 °C leaving 41.37% as residue. Char yield can be used as criteria for evaluating limiting oxygen index (LOI) of the polymers in accordance with Van Krevelen and Hoftyzer equation [44]. LOI = 17.5 + 0.4 CR where CR = chars yield. The LOI values of all polymers calculated based on their char yield at 700 °C was higher than 28. On the basis of LOI values, such macromolecules can be classified as self-extinguishing polymers. Polyamide **9** exhibited three endothermic decomposition peaks at 320 °C, 425 °C and 525 °C, respectively, which clearly demonstrated the highest thermal stability compared to analogues **10** and **12** in which both demonstrated one endothermic peak at 425 °C and 320 °C, respectively. Interestingly, thermal analyses of polyamides series **17**–**20** demonstrated similar thermal stability up to 700 °C due to the increase of the aromatic character and/or the presence of strong polar groups such as sulfonyl and ether linkages. Polyamides **18** and **19** exhibited two similar endothermic decomposition peaks at 200 °C and 425 °C while the analogue **20** demonstrated only one major endothermic peak at 425 °C. 

**Table 2:** Thermoanalytical and the kinetic parameters of polymers **9**, **10**, **12** and **17**–**20**.

Cpd. No.	Stage	TG (°C)	% Wt loss	LOI ^a^	T ^b^	*E*_a_^c^	A (S^−1^) × 10^−4^	ΔH* ^c^	ΔS* ^c^	ΔG* ^c^
**9**	I	200–700	58.60		320	6.72	0.47	1.78	−329.1	199.9
	II	Residue	41.40	34.1	425	22.34	0.72	16.53	−327.0	244.7
					525	86.16	1.40	79.52	−322.3	336.6
**10**	I	200–700	58.19	34.2	425	19.62	1.97	17.04	−317.2	184.9
	II	Residue	41.81							
**12**	I	200–700	58.63	41.1	320	82.97	3.72	78.20	−311.8	256.8
	II	Residue	41.37							
**17**	I	200–700	68.00	30.3	200	74.79	5.18	70.85	−317.7	221.1
	II	Residue	32.00		320	182.96	1.49	178.02	−309.0	361.6
					425	49.09	3.11	43.28	−314.9	263.1
**18**	I	200–700	46.82	38.8	200	143.60	1.81	139.66	−316.1	289.2
	II	Residue	53.18		425	90.69	3.53	84.88	−313.7	303.8
**19**	I	200–700	40.98	41.1	200	52.34	1.44	48.40	−322.3	200.8
	II	Residue	59.02		425	42.50	2.05	36.74	−318.3	257.3
**20**	I	200–700	52.29	36.6	425	133.87	8.40	128.06	−310.8	345.0
	II	Residue	47.71							

**^a^** Limiting oxygen index; **^b^** The peak temperature from the DTG charts; **^c^** Values are in kj/mole.

The thermodynamic parameters of decomposition processes of polymers, namely, activation energy (*E_a_*), enthalpy (ΔH*), entropy (ΔS*), and Gibbs free energy change of (ΔG*) were evaluated graphically by employing the Coats-Redfern method [45]. This method, reviewed by Johnson and Gallagher [46] as an integral method assuming various orders of reaction and comparing the linearity in each case to select the correct order by using equations (i) and (ii):


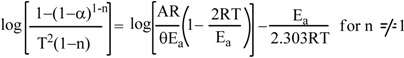
(i)


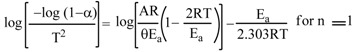
(ii)

where *α* is the fraction of sample decomposed at time *t*, *T* is the derivative peak temperature, *A* is the frequency factor, *E*_a_ is the activation energy, *R* is the gas constant, *θ* is the heating rate, and (1 *−* (2*RT*/*E*_a_)) ≅ 1. A plot of log [−log (1 − *a*)/*T*^2^] *versus* 1/*T* gives a slope from which the *E*_a_ was calculated and *A* (Arrhenius factor) was determined from the intercept. The correlation coefficient R was computed by using the least square method for the above equations. The values of R^2^ were nearer to unity in most cases. Linear curves were drawn for different values of n equal 0, 0.33, 0.5, 0.66 and 1. The values of n, which gave the best fit, were chosen as the order parameter for the decomposition stage of interest. The entropy of activation was calculated [47] using equation (iii):


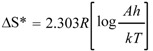
(iii)

where *h* and *k* stand for the Plank and Boltzmann constants, respectively, and *T* is the peak temperature from the DTG curve. The enthalpy of activation ΔH* and the free energy of activation ΔG* are calculated using equations (iv) and (v), respectively.


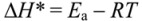
(iv)


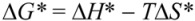
(v)

The kinetic data obtained from the nonisothermal decomposition of the prepared polymers **9**, **10**, **12** and **17**–**20** are given in Table 2. Activation energy values (*E*_a_) of polyamides **9** demonstrated higher E_a_ values compared to their analogues **12** and **10**, respectively. Activation energy values (E_a_) of other polyamides were in the order **17** > **18** > **20** > **19**, respectively, and thus indicating different degradation mechanism in all compounds. From the *E*_a_ values, one can conclude that the water molecules are easily eliminated from all ligands and the energies of activation for the second stages of decomposition are higher than that of the first stage. According to the kinetic data obtained from DTG curves all polymers have negative entropy (ΔS*), which indicates ordered systems and more ordered activated states that may be possible through the chemosorption of other light decomposition products [48]. The positive sign of the free energy of decomposition (ΔG*) of the investigated polymers indicated that the free energy of the final residue is higher than that of the initial compound and all the decomposition steps are non-spontaneous processes. Additionally, kinetic parameters showed that activation energies of decomposition, and hence the rate of decomposition, and the enthalpy (ΔH*) the polymers follow the sequence **9** > **12** > **10** and **17** > **18** > **20** > **19**, respectively, and the positive values of ΔH* means that the decomposition processes are endothermic. 

### 2.3. Biochemical Findings

**Figure 3:**
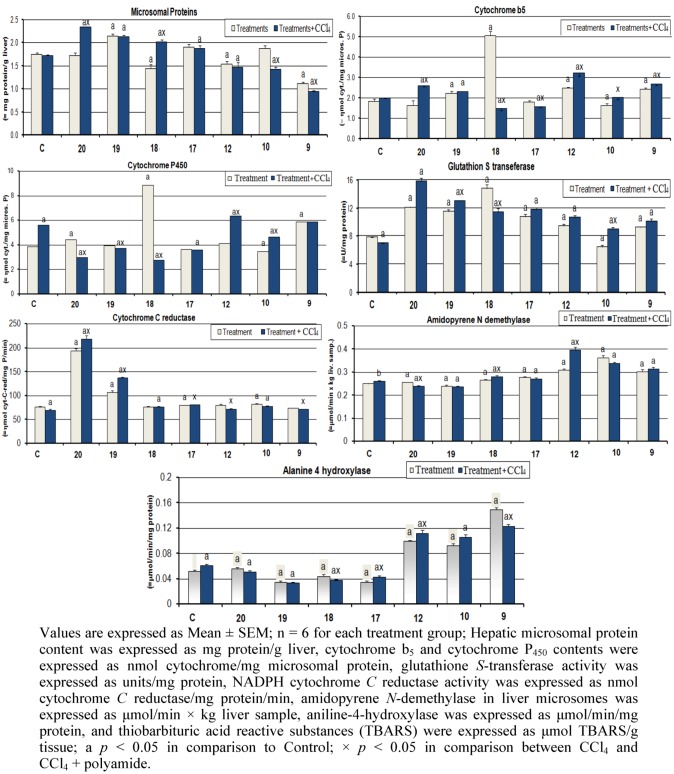
Effect of polyamides and carbon tetrachloride on drug metabolizing enzyme activities, and TBARS levels of male rat liver.

The effects of the prepared aramide nanoparticles and carbon tetrachloride on drug metabolizing enzyme activities, and TBARS levels of male rat liver are presented in Figure 3. Intraperitoneal administration of CCl_4_ (1 mL/kg) insignificantly affects the hepatic content for microsomal protein, the same trend was observed in **10**, **17** and **20**. Pretreatment of rats with **17**–**20** prior to the administration of CCl_4_ significantly increased the hepatic content of microsomal protein by 7%, 21%, 15% and 34%, respectively, compared to the control group. The opposite trend was observed in rats pretreated with **9**, **10** and **12**, where this content significantly decreased by −46%, −19% and −16%, respectively, compared to the control group. The hepatic content of cytochrome b_5_ in rats treated with CCl_4_ was unchanged. Pretreatment of rats with **9**, **10**, **12**, **18** and **20** prior to the administration of CCl_4_ induced cytochrome b_5_. Cytochrome P_450_ was significantly induced by CCl_4_ treatment, however, **18**–**20** reduced P_450_ content by −3%, −28% and −22%, respectively, compared to the control group. Liver GST activity was significantly reduced by CCl_4_ treatment; meanwhile, polyamides induced GST activity compared to the control group. The activity of amidopyrene *N-*demethylase, aniline 4*-*hyroxylase and TBARS levels significantly induced in CCl_4_ group by 4%, 17% and 27%, respectively, compared to the control group while treatment with polyamides reduced the alteration caused by CCl_4_. The opposite trend was observed for NADPH cytochrome *C-*reductase.

A protein involved in the architecture and also in the physiology of the cell seems to occupy a key role in the cell metabolism [49]. In the present study the induction in protein levels in rats pretreated with polyamides **17**–**20** prior to the administration of CCl_4_ indicates an acceleration of protein anabolism during CCl_4_ intoxication. This induction may be one of the defense mechanism of the experimental animals against oxidative stress induced by CCl_4_ intoxication. The rate-limiting step in the activation and detoxification of toxic compounds is dependent on the rate of reduction of cytochrome P_450_-substrate complex, which in turn is dependent on the activation and turnover rates of NADPH cytochrome *C-*reductase, cytochrome b_5_ and on the total cytochrome P_450_ content [50]. Administration of polyamides as repeated dose prior to the administration of CCl_4_ was found to increase the hepatic content of cytochrome b_5_ and NADPH cytochrome *C-*reductase activity. This induction could be one of the defense mechanisms of the experimental animals to increase the rate of reduction of cytochrome P_450_-substrate complex because the total hepatic content of cytochrome P_450_ decreased in these groups. 

Pretreatment of rats with polyamides prior to the administration of CCl_4_ was found to decrease the activity of amidopyrene *N*-demethylase. Inhibition of cytochrome P_450_ content and amidopyrene N-demethylase activity may play a significant role in the reduction of tumorgenicity and carcinogenicity of N-nitroso compounds [51]. CCl_4_ induced liver aniline 4-hydroxylase activity. On the other hand, polyamides ameliorate the action of CCl_4_. In accordance with the present study it has been found that the activity of aryl hydrocarbon hyroxylase (AHH) was decreased after pretreatment of rats with repeated doses of garlic and other free radical producer (CCl_4_) as a single dose. 

Lipid peroxidation is one of the principal causes of CCl_4_-induced liver injuries mediated by free radical derivatives of CCl_4_. The antioxidative activity of some substances and inhibition of free radical generation is important in protecting the liver [52] from CCl_4_ induced damage. CCl_4_ increased TBARS level after single dose treatment, the recovery in TBARS level due to pretreatment of rats with polyamides could be one of the protective mechanisms against the toxicity caused by CCl_4_ and probably other compounds. 

## 3. Experimental

### 3.1. Materials

*S-*Valine, 5-aminoisophthalic acid, *m*-phenylenediamine, *m*-aminophenol, 2,6-diaminopyridine, 4,4'-diaminodiphenylsulfone, 4,4'-diaminodiphenylether, 4,4'-diaminodiphenylmethane, benzidine (all Aldrich, Taufkirchen, Germany), and the solvents 1,4-dioxane (Aldrich), *N,N*-dimethylacetamide (DMAc, Aldrich), 1-methyl-2-pyrrolidone (NMP) (Fluka), dimethylsulfoxide (DMSO, Aldrich) were used as purchased without purification. Carbon tetrachloride was obtained from Merck (Darmstadt, Germany). Reduced glutathione (GSH), 1-chloro-2,4-dinitrobenzene (CDNB), reduced nicotinamide adenine dinucleotide phosphate (NADPH), amidopyrene, cytochrome C, thiobarbituric acid and all other chemicals were purchased from Sigma Chemical Company (St. Louis, MO, USA). Use of carbon tetrachloride was approved by the Animal Care Committee and met all guidelines for its use.

### 3.2. Measurements

Infrared spectra (IR, KBr pellets; 3 mm thickness) were recorded on a Perkin-Elmer FTIR 1650 Infrared Spectrophotometer. All spectra were recorded within the wave number range of 500–4,000 cm^−1^ at 25 °C. Absorption spectra were measured with a UV 500 UV-vis spectrometer at room temperature (rt) in DMSO with a polymer concentration of 2 mg/10 mL. Differential thermo gravimetric (DTG) analyses were carried out in the temperature range from 20 °C to 400 °C in a stream of nitrogen atmosphere by Shimadzu DTG 60H thermal analyzer. The experimental conditions were: Platinum crucible, nitrogen atmosphere with a 30 mL/min flow rate and a heating rate 10 °C/min. Differential scanning calorimetry (DSC-TGA) analyses were carried out using SDT-Q600-V20.5-Build-15 at the Institute of Graduate Studies and Research, Alexandria University. Inherent viscosities (η_inh_) were measured at a concentration of 0.5 g/dL in DMSO at 30 °C by using an Ubbelohde viscometer. Elemental analyses were performed at the Microanalytical Unit, Cairo University. The morphologies of polymer nanoparticles were observed by Scanning Electron Microscope (SEM) (JEOL-JSM5300), at the E-Microscope Unit; Faculty of Science, Alexandria University. The samples were sonicated in de-ionized water for 5 min and deposited onto cover glass and allowed to air-dry and sputter-coated with gold prior to examination. Biological studies were performed at the department of environmental studies, Institute of Graduate Studies and Research, Alexandria University. 

### 3.3. Synthesis of 2-Phthalimidyl-3-methyl Butyric Acid Chloride (**4**) [19]

A mixture of phthalic anhydride (3.00 g, 20.27 mmol) and *S*-valine (**3**, 2.61 g, 22.30 mmol) in DMF (30 mL) was stirred under reflux for 1 h. The solvent was removed under reduced pressure and to the residue a cold mixture of water (50 mL) and concentrated hydrochloric acid (5 mL) was added. The solution was then stirred for 2 h. A white precipitate was formed, filtered off and dried, to give 4.0 g (85% yield) of the known *2-phthalimidyl-3-methyl butyric acid*, m.p. 112–113 °C. 

Suspension of compound *2-phthalimidyl-3-methyl butyric acid* (4.0 g, 16.91 mmol) in SOCl_2_ (30 mL) was refluxed on boiling water bath. After 10 minutes, a clear solution was obtained and heating was continued for additional 2 h. Excess of the solvent was removed under vacuum and *n*-hexane (20 mL) was added. The resulting white solid was collected by filtration and dried *in vacuo* to give 4.0 g (93% yield) of **4**, m.p. 82–83 °C.

### 3.4. Synthesis of 5-(2-Phthalimidyl-3-methyl butanoylamino)isophthaloyl Chloride (**6**)

Into a 25 mL round-bottomed flask fitted with a magnetic stirrer was placed a solution of *5-amino-isophthalic acid* (**5**, 2.72 g, 15.02 mmol) in DMAc (25 mL). While stirring, a solution of the acid chloride **4** (4.0 g 15.06 mmol) in DMAc (25 mL) was added dropwise and the mixture was stirred for 10 h (overnight) at room temperature. Triethylamine (TEA, 0.52 mL) was added to this mixture and heated at 80 °C for an additional 4 h. The solution was poured into a mixture of water (250 mL) and concentrated hydrochloric acid (5 mL). The white precipitate was collected by filtration and washed thoroughly with water and dried in a vacuum oven at 60 °C for 10 h, to give 3.5 g of the corresponding diacid (56.8% yield), m.p. >250 °C. A suspension of the resulting diacid (2.00 g, 4.87 mmol) in SOCl_2_ (20 mL) was refluxed on a boiling water bath. After 30 minutes, a clear solution was obtained and heating was continued for additional 4 h. Excess of the solvent was removed under vacuum and *n*-hexane (20 mL) was added. The white solid was collected by filtration and dried *in vacuo* to give 2.00 g (92% yield) of **6** as a yellow solid, m.p. 128–129 °C. 

### 3.5. Polymer Particles Synthesis (General Method)

The diacid chloride **6** (0.5 mmol) and the appropriate diamine **7**, **8**, **13**–**16** or *m*-aminophenol **11** (0.5 mmol) were each dissolved in dioxane (50 mL). Distilled water (15 mL) was added to the solution of the diamine followed by the addition of the entire acid chloride solution at once. The resulted turbid solution was ultrasonicated at 42 KHz in a water bath for a period of 30 min. The polymer colloidal solution was extracted by centrifugal separation for 15 min. at 15,000 rpm and the resulted precipitate were carefully washed with methanol and water to purify the product of any unreacted monomer. The polymer samples were then dried at 100 °C for 10 h then kept in a vacuum desiccator. 

### 3.6. Biological Studies

The cytochrome P_450_ enzymes are responsible for the oxidation of xenobiotic chemicals including drugs, pesticides and carcinogens. These enzymes include cytochrome P_450_, cytochrome b_5_, NADPH cytochrome *C*-reductase, amidopyrene *N*-demethylase and aniline *4*-hyroxylase (phase I). In addition to the above enzymes, agents involved in phase II drug metabolism e.g. glutathione *S*-transferase (GST) as well as free radicals, detected as thiobarbituric acid-reactive substances (TBARS). Changes in the activities of these enzymes were studies in the liver microsomes of rats treated with antioxidant (polyamides) as repeated doses prior to the administration of a single dose of carbon tetrachloride (CCl_4_). 

#### 3.6.1. Animals and Treatments

Ninety six male Sprague Dawley rats, six weeks old weighing 100–120 g were obtained from the National Research Institute, Cairo, Egypt and acclimatized for two weeks prior to the experiments. They were assigned to 16 groups and housed at Universal galvanized wire cages at room temperature (22–25 °C) and in a photoperiod of 14 h light/10 h dark per day. Animals received standard laboratory balanced commercial diet and water *ad libitum*.

#### 3.6.2. Experimental Design

Animals were randomly allocated into 16 groups (six rats/group) and treated as follows: group (1) served as a control and was given deuterated dimethyl sulphoxide (DMSO-*d_6_*) at the same volume as the vehicle (ip), in order to standardize the stress stimulation due to the capture and medicine application; group (2) received a single injection of CCl_4_ in corn oil (ip) at dose level of 1 mL/kg [53] during the last two weeks (*i.e*., day 15), groups from 3 to 16 were treated with the polyamides (ip) for two weeks (three times a week; 25 mg/kg) and then groups 4, 6, 8, 10, 12, 14, 16 were administered CCl_4_ during the last two weeks. After 4 days of CCl_4_ injection, the samples were collected.

#### 3.6.3. Tissue Preparations and Assays

##### 3.6.3.1. Preparation of Liver Microsomes

Rats were fasted 24 h prior to each designated time point and then sacrificed by cervical dislocation. The abdominal cavity was opened immediately, the liver was removed, washed with cold 0.1 mol phosphate buffer, pH 7.4, weighed and chilled on ice. All the following procedures were carried out under cold conditions. A 33% (W/V) crude homogenate was prepared in 0.1 mol phosphate buffer, pH 7.4 by homogenization with a Teflon pestle, using five strokes. The crude homogenate was then centrifuged at 11,000 ×g for 20 min at 4 °C to remove the intact cells, nuclei and mitochondria. The supernatant solution was subsequently centrifuged at 105,000 ×g for 60 min at 4 °C to sediment the microsomal pellet. The pellet was re-suspended in 0.1 mol phosphate buffer, pH 7.4, kept in an ice bath and used as the enzyme source.

##### 3.6.3.2. Protein Determination

The protein concentration of the hepatic microsomal fractions was determined by the method of Lowery *et al.* [54].

#### 3.6.4. Enzyme Assays

Liver microsomal cytochrome P_450_ and b_5_ were determined according to Omura and Sato [55], using a molar extinction coefficient 91 cm^−1^ m mol^−1^ for P_450_ and 185 cm^−1^ m mol^−1^ for cytochrome b_5_. The activity of microsomal NADPH-cytochrome-C-reductase was assayed according to the method of Williams and Kamin [56]. The rate of reduction of cytochrome C was measured at zero and 30 seconds after the addition of NADPH at wavelength 550 ηm. The activity of this enzyme was calculated by using an extinction coefficient of 21 cm^−1^ m mol^−1^.

Glutathione-*S*-transferase activity was assayed according to the method of Habig *et al.* [57]. A unit of enzyme activity is defined as the amount of enzyme that catalyzes the formation of 1 μmol of 1-chloro-2,4-dinitrobenzene (CDNB) conjugate/mg protein/min under the assay conditions. The CDNB conjugate was measured spectrophotometrically at 340 ηm. Calculations were made using a molar extinction coefficient of 9.6 cm^−1^ m mol^−1^.

The activity of amidopyrene *N*-demethylase was measured according to Nash [58]. The method is based on measurement of the concentration of formaldehyde produced by oxidative *N*-demethylation of amidopyrene in microsomes. Formaldehyde was determined spectrophotometrically from changes in the color intensity of the supernatant at 412 ηm. The enzymatic activity was then expressed as μmol of formaldehyde/min × kg liver sample.

The activity of aniline 4-hydroxylase was measured according to Kato and Gillette [59]. The color developed was measured spectrophotometrically at 630 ηm.

Thiobarbituric acid reactive substances (TBARS) were measured according to the method described by Tapel and Zalkin [60]. The color intensity of the TBARS reactants was measured at 532 ηm and a molar extinction coefficient of 156,000 cm^−1^ mol^−1^ was used for calculation of the concentration.

#### 3.6.5. Statistical Analyses

Mean and standard error values were determined for all the parameters and the results were expressed as mean ± standard error for six rats in each group. The data were analyzed using a one-way analysis of variance (ANOVA). The student-Newman-keuls test was used to compare the treated and control groups and the significance is given as *p* < 0.05. 

#### 3.6.6. Clinical Findings

No clinical sings of CCl_4_ poisoning were evident among rats of the treatment groups. Farther, no mortality occurred in any of the treatment groups during the experimental period.

## 4. Conclusions

New nanoscale chiral aramides containing *N*-phthaloyl valine moieties in the side chain were successfully prepared and characterized. Polyamide nanoparticles **9** and **10**, derived from *m*-phenylenediamine and 2,6-diaminopyridine, respectively, were obtained with average diameters of 106 nm, while polyesteramide nanoparticles **12**, derived from *m*-aminophenol, produced under the same reaction conditions were obtained with larger diameters (200 nm). Polymeric particles **9** and **12** were obtained as well-separated spherical nanoparticles while aggregated particles of the aramide-containing pyridine **10** form a thin layer. The formation of such thin layer is attributed to the molecular self assembly via H-bond directed organization of molecular precursors. Based on the flexible linkage type, the average diameters of the nanoparticles-containing methylene **18**, ether **19** and sulfone **20** linkage were 260 nm and 120 nm, respectively, as judged by SEM photographs. The inherent viscosities of the nanoparticles were in the range of 0.58–0.70 dL/g and the particles exhibited similar solubility behaviors in different organic solvents. The structures were carefully characterized by different techniques such as IR, UV, elemental analyses. The thermal properties were evaluated by TG/DTG and DSC techniques and results revealed that the prepared polymers have high thermal stability. Structure-thermal property correlation based on changing the diamine monomer, as a single structural modification, is also studied. The limiting oxygen index (LOI) values of polymers calculated based on their char yield at 700 °C clearly revealed that the reported macromolecules can be classified as self-extinguishing polymers.

The effects of the aramide nanoparticles and carbon tetrachloride on drug metabolizing enzyme activities and TBARS levels of male rat liver were studied. Pretreatments of rats with repeated doses of the polyamides prior to the administration of CCl_4_ not only decreased the hepatic content of cytochrome P_450_, amidopyrene *N*-demethylase, aniline-*4*-hyroxylase, but also induced of the hepatic content of cytochrome b_5_ and NADPH cytochrome *C*-reductase. Repeated doses of the tested compounds could reduce the toxic effects exerted by CCl_4_ upon the liver, and probably other organs, through inhibition of cytochrome P_450_ system that activates CCl_4_ into its active metabolite, trichloromethyl radical. Moreover, inhibition of cytochrome P_450_ system could also reduce the toxic and carcinogenic effects of chemical carcinogens. 
